# Humans as Long-Distance Dispersers of Rural Plant Communities

**DOI:** 10.1371/journal.pone.0062763

**Published:** 2013-05-02

**Authors:** Alistair G. Auffret, Sara A. O. Cousins

**Affiliations:** Landscape Ecology, Department of Physical Geography and Quaternary Geology, Stockholm University, Stockholm, Sweden; New York State Museum, United States of America

## Abstract

Humans are known for their capacity to disperse organisms long distances. Long-distance dispersal can be important for species threatened by habitat destruction, but research into human-mediated dispersal is often focused upon few and/or invasive species. Here we use citizen science to identify the capacity for humans to disperse seeds on their clothes and footwear from a known species pool in a valuable habitat, allowing for an assessment of the fraction and types of species dispersed by humans in an alternative context. We collected material from volunteers cutting 48 species-rich meadows throughout Sweden. We counted 24 354 seeds of 197 species, representing 34% of the available species pool, including several rare and protected species. However, 71 species (36%) are considered invasive elsewhere in the world. Trait analysis showed that seeds with hooks or other appendages were more likely to be dispersed by humans, as well as those with a persistent seed bank. More activity in a meadow resulted in more dispersal, both in terms of species and representation of the source communities. Average potential dispersal distances were measured at 13 km. We consider humans capable seed dispersers, transporting a significant proportion of the plant communities in which they are active, just like more traditional vectors such as livestock. When rural populations were larger, people might have been regular and effective seed dispersers, and the net rural-urban migration resulting in a reduction in humans in the landscape may have exacerbated the dispersal failure evident in declining plant populations today. With the fragmentation of habitat and changes in land use resulting from agricultural change, and the increased mobility of humans worldwide, the dispersal role of humans may have shifted from providers of regular local and landscape dispersal to providers of much rarer long-distance and regional dispersal, and international invasion.

## Introduction

With habitat loss and fragmentation representing the largest challenge for biodiversity worldwide [1], the long-distance dispersal of species and populations between suitable habitat patches is a vital mechanism in retaining biodiversity at multiple scales [2], [3]. Long distance dispersal events often occur via a non-standard dispersal vector [4]–[6], and exploring these is important for the understanding of plant communities worldwide in the face of habitat destruction and climatic change.

In human-dominated landscapes, people have a major influence not only on the distribution, but also on the dispersal of plant species [7]. Human-mediated dispersal in these environments can be either intentional or unintentional. The imprint on the vegetation of intentional, or directed dispersal, such as via grazing animals moved by land managers can be assessed due to the regularity and predictability of the vector movements [8], [9]. On the other hand, unintentional dispersal such as via motor vehicles is more stochastic by nature [10], [11], but investigations of the types of seeds dispersed and an assessment of dispersal distance are useful given the importance of non-standard dispersal vectors in community dynamics.

Unintentional dispersal of seeds attached to the clothes and shoes of humans (anthropochory) has been known about for some time [12]–[14], but only recently has its importance been appreciated, and efforts been made to quantify the species dispersed and the distances travelled. Indeed, measured dispersal distances of seeds attached to humans walking have been found to exceed species’ normal dispersal distances [15], [16], while other studies have investigated seeds dispersed at the international and continental scale [17], [18]. Through this human-mediated dispersal, the increased mobility of humans worldwide during the past centuries has aided the spread of invasive plant species into fragile ecosystems and biomes at an ever increasing rate since the Middle Ages [19].

Due to the perceived risks associated with human-mediated dispersal, investigations have been concentrated in ecologically sensitive regions, with studies in continental Europe rare and largely qualitative [13], [14]. In this region, human activity over long time periods has produced semi-natural grasslands which rank among the world’s most species-rich habitats [20]. Agricultural change during the 20th century has dramatically reduced the size and number of these grasslands [e.g. 21,22], directly threatening biodiversity, while also changing the roles of traditional seed dispersers such as free-roaming grazing livestock by restricting their movement [23]. This has led to a further isolation of dispersal limited plant communities at risk of local extinction [24], [25]. The mechanisation of agriculture has also contributed to net rural-urban migration, and a fall in the number of humans working and living in rural landscapes worldwide [26]. The reduction of rural human populations may well have also represented a reduction in an effective seed disperser, and today, the remaining rural population might still provide infrequent, random long-distance dispersal events in the landscape.

As grassland extent and rural populations have declined, volunteer conservationists and citizen scientists can provide an important service in both the maintenance and monitoring of biodiversity [27]. Using mainly citizen collected data, we investigate human-mediated seed dispersal in the rural landscape, collecting seeds attaching to volunteers cutting species-rich meadows and comparing these with the local available species pool. As hay-cutting events occur at a time of high seed availability, this allows for an approximation of the potential seed dispersal during the whole growing season. For the first time, we compare human-dispersed species with those in the local species pool, allowing us to ask how dispersed species reflect plant communities, and which traits select for human-mediated dispersal. Potential dispersal distances are measured, and comparing dispersed species with threatened, protected and invasive species, we discuss the role of this type of human-mediated dispersal in the rural landscape.

## Materials and Methods

### Site Selection and Source Communities

**Figure 1:**
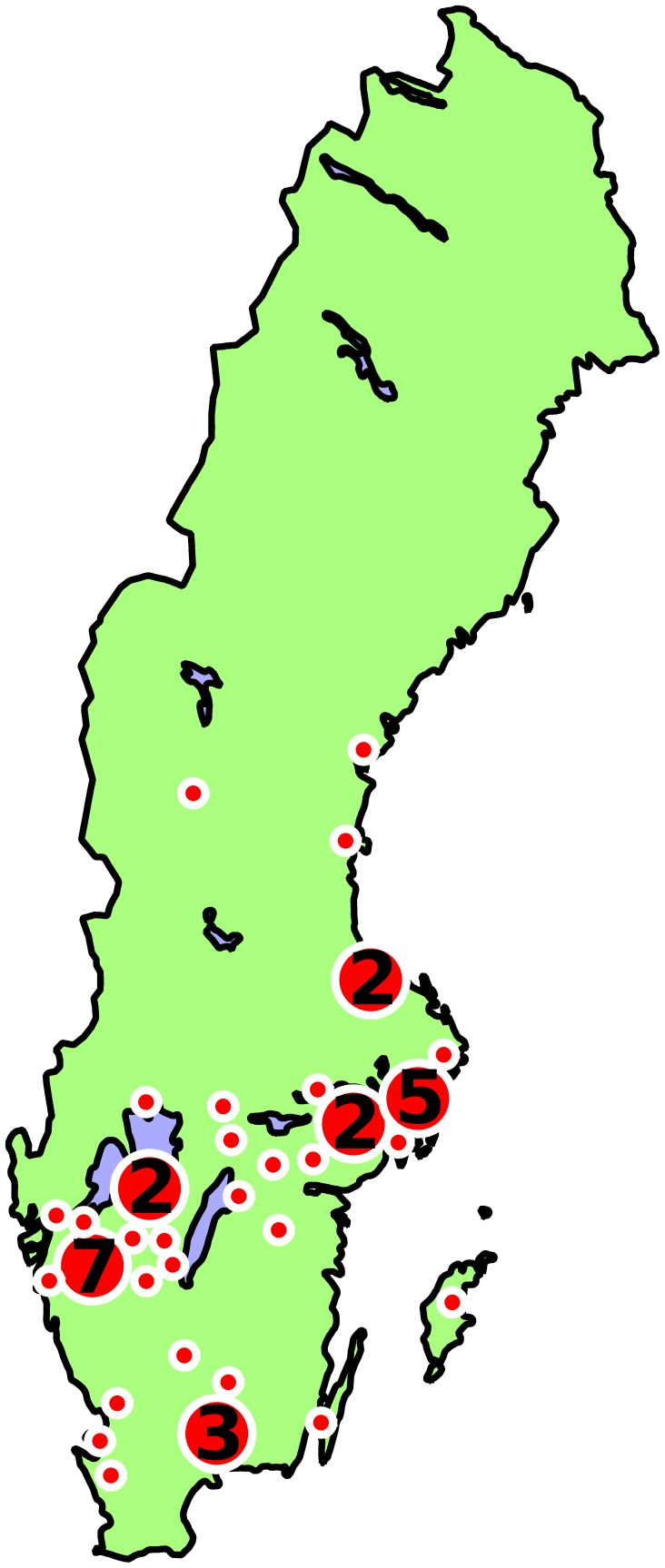
Locations of 48 meadows in Sweden from which human-mediated dispersal samples were collected. Numbers in circles represent areas where several meadows are located close to one another.

This study was carried out in meadows managed by local branches of the Swedish Society for Nature Conservation (SSNC - Swedish: *Naturskyddsföreningen*). All branches across Sweden who manage a meadow were asked by the corresponding author to participate in this study. Thirty-eight groups agreed to take part in the study, managing a total of 48 meadows across Sweden (see Figure 1, Appendix S1). The SSNC branch is responsible for managing each meadow, but land ownership is spread between privately-owned agricultural estates, the state as part of national nature reserves, and meadows on land owned by the SSNC itself. The meadows are small fragments of species-rich grassland habitat (<1 ha, mown area often much smaller), with a history as meadow or unimproved pasture, though some may have been abandoned for a period of time before the SSNC began managing the site. Today, they are managed by clearing in the spring and traditional hay-cutting in the late summer. All meadows are valuable patches of grassland habitat; thirty-eight of the meadows are part of areas listed in the Swedish government’s survey of valuable semi-natural pastures and meadows 2002–2004 (TUVA database. Available at: http://www.sjv.se/tuva. Accessed: 2012 Dec 10), of which 25 are also designated EU Natura 2000 areas (Available at: http://natura2000.eea.europa.eu. Accessed: 2013 Jan 10). The continued grassland management by the SSNC groups is essential for maintaining the ecological value of all sites. Those meadows which are not recognised above are probably too small to be considered, such as those on small areas of land owned by the SSNC.

We were able to collect the available species pool (presence/absence) for 36 of the meadows. Nineteen species lists came directly from the SSNC group, seven were extracted from the Swedish Species Gateway (*Artportalen.* Available: http://www.artportalen.se/. Accessed: 2012 Dec 10), five from local or regional plant atlases, while we complemented the above with inventories of five further meadows in the vicinity of Stockholm. Although the inventories come from a range of sources, we only used those which we considered as coming from a reliable botanical source. See Appendix S1 for details.

### Seed Collection and Questionnaires

Information about the project, pre-paid envelopes, instructions and questionnaires were sent to the organiser of the hay-cutting to hand out to willing participants on the day. Participants were asked that on returning home after the hay-cutting, they should remove their outer clothes, shaking them and brushing them down, emptying pockets, picking visible plant material and emptying and banging together footwear. All resulting material was placed in one plastic bag and posted to the corresponding author. Participants also provided their postcode to allow for an estimation of dispersal distance from the meadow site. In total, 214 samples were received from the 48 meadows, each meadow providing between one and twelve samples. Samples were examined under a microscope, and seeds were identified to species level where possible, but in some cases identification was only possible to genus level and grouped as aggregates (hereafter counted as “species”). See Appendix S2 for details of aggregate species. Eighty-three seeds of the family Poaceae could not be identified to the genus level, and were ignored. Seeds that were clearly unviable (empty) were not counted, but in general seeds were not checked for viability.

### Ethical Considerations

The study did not involve a physical operation on the subjects, involve any sensitive information or remove biological material from the subjects which could be traced to the individual. Therefore, it was not covered by Swedish law 2003∶460 concerning ethical hearings requirements in research, and no permissions were sought from an ethics committee. Samples were sent anonymously, subjects were aware of the research project in which they were participating, and consent was inferred by the posting of samples to the corresponding author by each subject. The investigation was conducted within the authors’ country of residence.

The Swedish Society for Nature Conservation is a nationally recognised charity, and local groups have agreements with landowners, local councils and/or county board to manage the meadow sites not owned by the group itself. All described activity in the meadows, and the removal of seeds from them, would have taken place regardless of our investigation. Therefore, as no additional activity took place on any meadow, no permits were required to carry out the seed dispersal part of the investigation. The ‘freedom to roam’ law in Sweden (Law 2010∶1408) ensures that everybody has the right to non-destructive access to nature, meaning that no permits were required for the meadow inventories carried out by those not associated with the SSNC group.

### Data Analysis

Three samples contained no seeds and were removed before analysis. Cryptogams, and dust-seed producing Orchidacaea were removed from the species pool data before analysis as they would not be detected in our seed samples (Appendix S3). The average per-person and total numbers of seeds and species were calculated, and the list of dispersed species (including positively identified species later grouped as aggregates) was compared to nationally protected species (Species Protection Act – Statute 2007∶845), red-listed species [28], nationally invasive species from the European Network on Invasive Alien Species (NOBANIS. Available: http://www.nobanis.org}. Accessed: 2011 Mar 28) and internationally invasive species from the Centre for Agricultural Bioscience International (CABI. Available: http://www.cabi.org/isc/}. Accessed: 2012 Nov 21). To examine the effect of number of samples on seed dispersal, individual records were grouped by meadow and converted to species presence-absence. Then, the effect of sample number per meadow on species richness, and the Bray-Curtis similarity of dispersed seeds and source communities were tested by linear regression.

The types of seeds dispersed by humans were investigated using six seed traits relating to temporal and spatial dispersal ability. Data for all species from the seed samples and the species pools regarding seed bank (persistent or transient), seed mass (mg), seed morphology (hooked, otherwise appendaged or not appendaged), seed number, seed releasing height (m) and seed terminal velocity (ms^−1^) were extracted from the LEDA traitbase [29]. Due to overdispersion in the trait data, a quasibinomial logistic regression was performed for each trait separately to test which traits were related to whether seeds were dispersed by humans or not.

Potential dispersal distances were estimated by calculating the Euclidean distance between the meadow site where the seeds attached, and the point representing each participant’s home postal code in Yahoo! Maps (Available: http://maps.yahoo.com/. Accessed: 2012 Nov 06), where the seeds were removed from the clothing. All statistical analyses were carried out using R 2.14.1 [30], with the additional packages mefa [31], vegan [32] and RdbiPgSQL [33]. PostGIS 1.5.3 [34] was used for the distance calculation.

## Results

**Figure 2:**
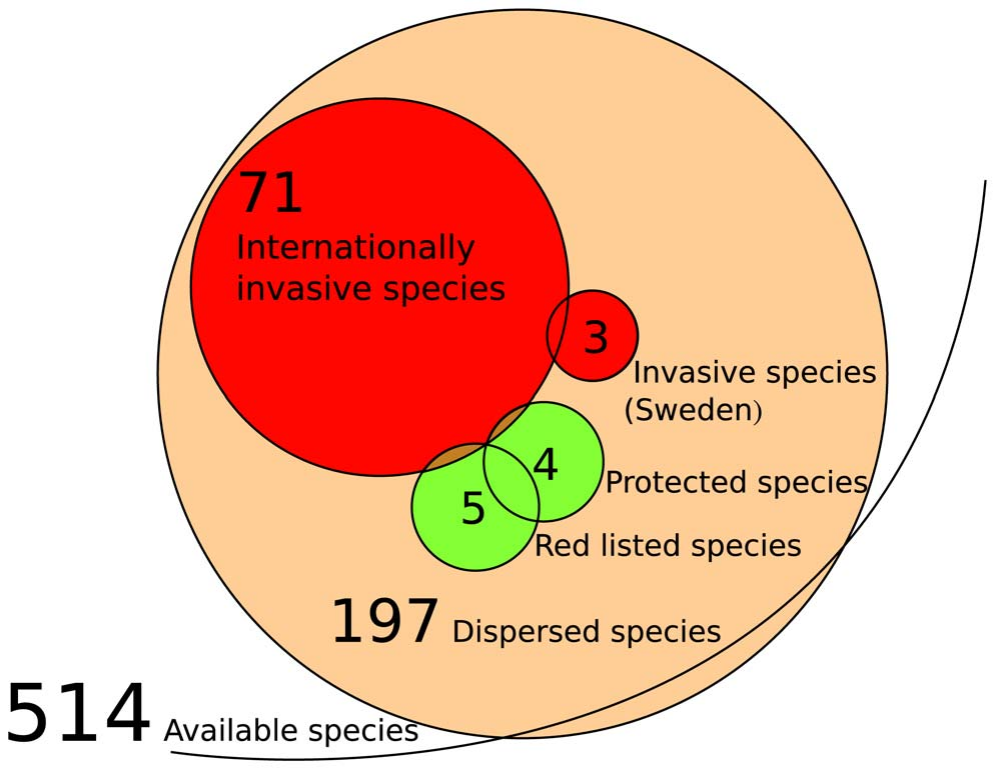
Groupings of species identified from samples of human-dispersed seeds from 48 Swedish meadows. For simplicity, invasive and protected species available in the plant communities are not considered.

A total of 24 354 seeds of 197 species were identified from the 211 seed-containing samples (Figure 2). Seed content was quite variable between samples, with a mean of 115±286 seeds and 11±7 species dispersed per sample. Four nationally protected species and five species on the red list were dispersed (two of the dispersed species were on both lists), while three invasive species were identified. Seventy-one dispersed species are listed as invasive in another country worldwide. The inventories gave an average of 104±50 plant species present in each meadow, with a total of 514 species across all meadows. Of these species, 34% were present in the seed samples. Twenty-four species identified in the seed samples were not present in any plant species inventory. A species list is available in Appendix S2.

**Figure 3:**
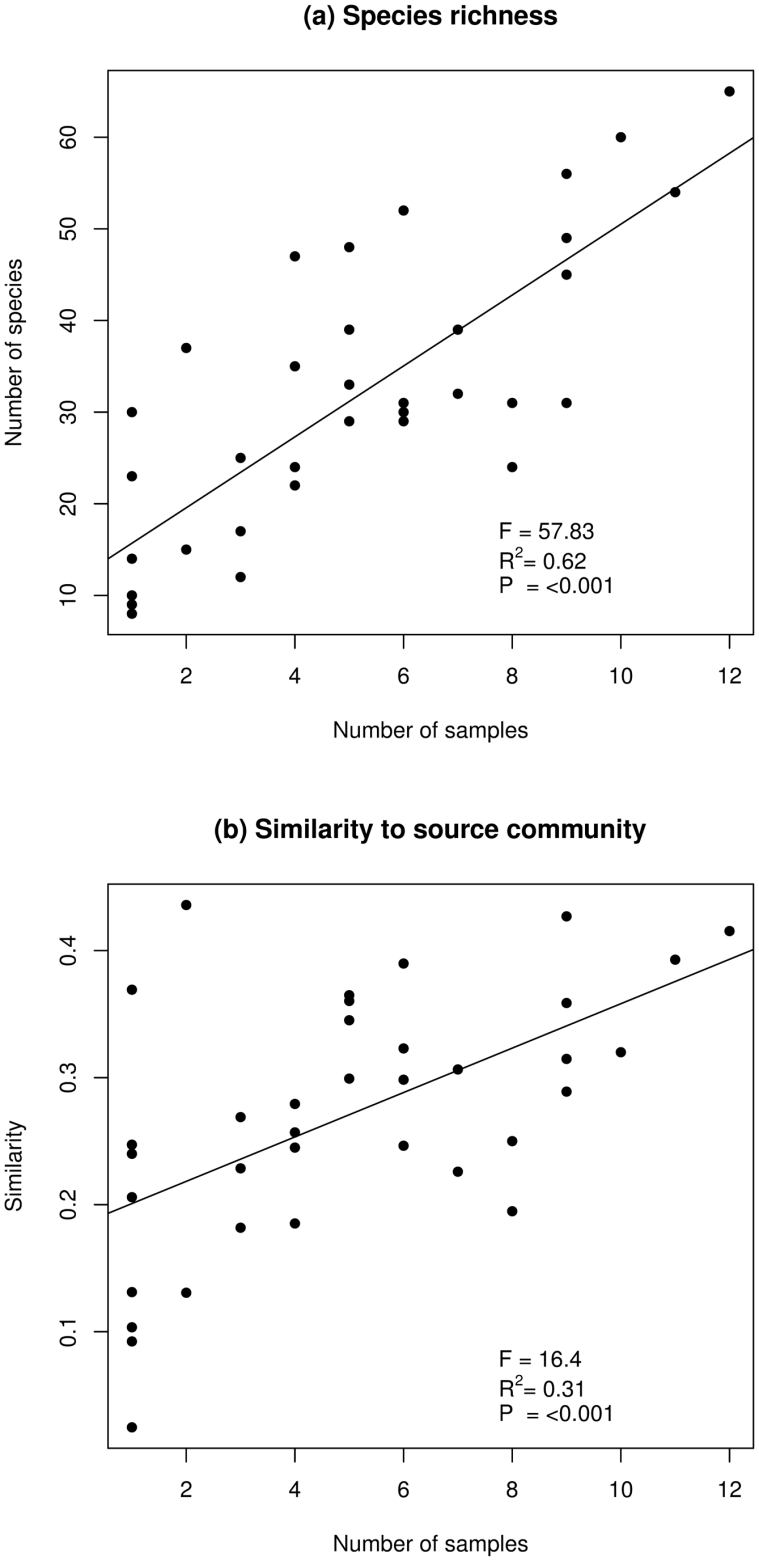
Effect of increasing sample size on human-mediated dispersal. Total species richness (a) and Bray-Curtis similarity to source community (b) of seeds dispersed with clothing and footwear of different numbers of volunteers cutting valuable meadows throughout Sweden.

More samples received from a meadow resulted in a significant increase in both the number of species dispersed, and the similarity between the seeds dispersed and plants available in the species pool (Figure 3). The logistic regression showed that both hooked and otherwise appendaged seeds, and those with a persistent seed bank were significantly associated with anthropochorous dispersal (Table 1). There was a marginally non-significant tendency for species with a low seed-releasing height to be dispersed. The distances between the meadows where seeds attached and the site of removal (potential dispersal distance) ranged from 1.3 km to 110 km, with an average distance of 13.4 km.

**Table 1:** Results of quasibinomial logistic regression comparing species traits dispersed by humans with those in the available species pool of 36 meadows.

	Coefficient	SE	P
**Seed bank persistence**	**1.96**	**0.31**	**<0.001**
Seed mass	−0.0096	0.0054	0.08
**Seed morphology**			
**- appendaged**	**0.87**	**0.20**	**<0.001**
**- hooked**	**1.70**	**0.57**	**0.003**
Seed number	− <0.001	<0.001	0.31
Seed release height	−0.053	0.027	0.052
Seed terminal velocity	0.022	0.083	0.79

A positive coefficient indicates a positive effect, and a negative coefficient a negative effect. A coefficient further from zero indicates a stronger effect. Significant traits are in bold.

## Discussion

We have identified humans as potentially effective long-distance seed dispersers in rural areas. All but three of the 214 samples of material from clothes and shoes contained seeds (99%), with many containing hundreds of seeds and several species. Seeds of approximately one-third of the available species of these valuable grassland habitats attached to the clothing of people working in them, including several protected or red-listed species, indicating that the majority of the seeds identified originated from the focal meadows. The over-representation of hooked and appendaged seeds shows that humans can disperse seeds just like any other animal in the landscape [35], [36], while the dispersal of seeds with persistent seed banks shows how humans can spatially disperse seeds usually associated with dispersal through time.

Our results offer an alternative context of human-mediated dispersal. Whereas the focus of seeds transported directly by humans is usually related to species invasions, we instead found a greater number of protected species than national invasives. Furthermore, the three invasive species identified are widespread, and were introduced to Sweden more than 250 years ago (NOBANIS. Available: http://www.nobanis.org. Accessed: 2011 Mar 28). We therefore do not consider dispersal on clothing as a major driver of invasive species today at the local and regional scale. We did however identify 71 species (36% of those dispersed) which are listed as invasive in other countries worldwide. This highlights the fact that one only needs to step on an aeroplane to change the context of human-mediated seed dispersal, and confirms the risks identified by those working in sensitive environments [17], [18].

More samples received from a meadow resulted in a greater number of species dispersed and a better representation of the source community. This is an intuitive result, but nevertheless demonstrates how unintentional and non-standard seed dispersal is moderated by the people moving and living in the landscape. Furthermore, the linear relationship between number of samples and the number of species and available-dispersed community similarity (Figure 3) indicates that given more activity, more dispersal would have taken place and a higher fraction of the available species would be represented in the samples. In the past, when rural populations were larger [26], humans might have been regular and effective seed dispersers, contributing to vegetation dynamics in rural landscapes. Dispersing the same kinds of seeds as the free-roaming livestock, the reduction in humans in the landscape may have exacerbated the dispersal failure evident in declining plant populations today [24]. In the modern rural landscape, temporary large rural populations are formed by tourists and summer residents, whose increasing activity is often linked to the spread of alien species [37]. Where invasive plant species are a lesser threat, increased human activity can be linked to the dispersal and higher turnover of native species [38].

The natural attachment to clothes of a meaningful proportion of available and target species is a significant finding. The long distances our seeds were transported is indicative of how increased human mobility has led to increased seed mobility. As most of the volunteers will have driven home after the hay-cutting, the measured potential dispersal distances were able to exceed those from more traditional vectors [39]. The fraction of available species dispersed by humans falls well within the ranges reported for livestock grazing semi-natural pasture [7], seen as more traditional seed dispersal vectors in rural areas, while Figure 3 indicates that a higher proportion may have been reached with more samples. Subsequent detachment of seeds is difficult to estimate [40], both in terms of distance and direction. However, studies in human-mediated dispersal which have examined detachment of experimentally attached seeds from shoes and clothing indicate that seeds do detach quite readily [15], [16], compared to grazing livestock on which seeds can become deeply buried in fur and effectively stuck to the animal [40]. In fragmented landscapes, the successful dispersal of plant species via humans between suitable habitat is probably quite small. However, as animal behaviour is implicated in the distance and direction of seed dispersal by frugivores [41], human-mediated dispersal of both invasive and valuable plant communities may well be directed by the behaviour of those active in rural areas.

Citizen science is inherently prone to bias [42], but in our case it has given us the unique opportunity to study human-mediated dispersal in similarly-managed habitats with reliably sourced species pool data. The need for adequate sample numbers means that human-mediated dispersal studies are themselves generally biased towards tourists and/or researchers [17], [18], [43] who have more predictable movement patterns in areas of ecological interest. It is more difficult to measure the everyday dispersal by humans in everyday landscapes, but here we have shown that people have the capacity to disperse seeds from a reasonable fraction of the plant communities around them. Coupled with the fact that almost all samples were found to contain seeds, our results indicate that humans have the capacity to provide rare, but disproportionately important non-standard long-distance dispersal events [4].

Harnessing the engagement of citizen scientists and volunteer conservationists, we have for the first time quantified seed dispersal directly by humans from a known species pool. This has allowed us to find that humans are capable seed dispersers like any other animal, transporting one-third of available species, and those with a range of different dispersal mechanisms. Species were dispersed which on the one hand are protected by national law and international biodiversity agreements, but on the other hand are invasive aliens in other countries under the same agreements. We believe that alternative contexts of human-mediated dispersal should be considered, dependent on the time of dispersal and the distance travelled. Not only does human-mediated dispersal provide a risk of invasive species entering fragile ecosystems, but it could also be a potential source of past and present connectivity and community build-up on more local and regional scales. From individuals in groups interested in conserving historical habitats to national governments protecting biodiversity, the capacity for humans and their associated vectors to mobilise seeds is of considerable ecological interest. With the fragmentation of habitat and urbanisation of rural populations resulting from agricultural change, the dispersal role of humans may have shifted from providers of regular local and landscape dispersal to providers of much rarer long-distance and regional dispersal, and international invasion.

## Supporting Information

Appendix S1 **Information regarding the 48 meadows used for the human-mediated dispersal study.**
(DOC)

Appendix S2 **Species of seeds dispersed by humans from meadows across Sweden.**
(DOC)

Appendix S3 **Plant species recorded in meadows but of which no seeds were dispersed.**
(DOC)
