# Supplementary material for: Humans as Long-Distance Dispersers of Rural Plant Communities
Source: PLoS One. 2013 May 2;8(5):e62763. doi: 10.1371/journal.pone.0062763 (PMC3642177; doi:10.1371/journal.pone.0062763)
Supplement: Appendix S1 — Information regarding the 48 meadows used for the human-mediated dispersal study. (DOC) [file pone.0062763.s001.doc]

**Appendix S1**. Information regarding the 48 meadows used for the human-mediated dispersal study. TUVA= Listed in the Swedish government's survey of valuable semi-natural pastures and meadows 2002-2004 (Available at: <http://www.sjv.se/tuva>. Accessed: 2012 Dec 10). N20000 = Designated as a EU Natura 2000 area (Available at: [http://natura2000.eea.europa.eu](http://natura2000.eea.europa.eu/). Accessed: 2013 Jan 10). SSNC = Swedish Society for Nature Conservation. Artportalen = Swedish Species Gateway (Available: <http://www.artportalen.se/>. Accessed: 2012 Dec 10)

.

| Group | Meadow | Location (SWEREF 99 TM) | | TUVA | N2000 | Samples (with seeds) | Inventory | Comments: |
| --- | --- | --- | --- | --- | --- | --- | --- | --- |
|  |  | x | y |  |  |  |  |  |
| Alingsås | Åsgärde | 368526 | 6425365 | 1 | 0 | 1 | 0 |  |
| Alingsås | Brobacka | 348523 | 6429010 | 1 | 1 | 1 | 1 | Artportalen. Inventory of meadow for regional plant atlas 1987. |
| Alingsås | Bryngels gärde | 353568 | 6409894 | 1 | 1 | 1 | 1 | Artportalen. Inventory of meadow for regional plant atlas 1993. |
| Alingsås | Damsjöås | 359208 | 6420564 | 1 | 0 | 1 | 0 |  |
| Alingsås | Långeviken | 356164 | 6410435 | 1 | 0 | 2 | 0 |  |
| Alingsås | Torstö | 347095 | 6422172 | 1 | 0 | 2 | 0 |  |
| Alingsås | Vrån | 359763 | 6416696 | 1 | 0 | 2 | 0 |  |
| Älvkarleby | Pagards äng | 633991 | 6717373 | 0 | 0 | 1 | 1 | Complementary inventory by N. Hedberg (Stockholm University) 2011. Species present in 10 1 m^2^ quadrats plus walk to find additional species. |
| Älvkarleby | Rotskärsängen | 633423 | 6723876 | 1 | 0 | 1 | 1 | Complementary inventory by N. Hedberg (Stockholm University) 2011. Species present in 10 1 m^2^ quadrats plus walk to find additional species. |
| Bjäre | Haralds äng | 366315 | 6248771 | 1 | 1 | 6 | 1 | SSNC inventory ca. 1995 |
| Finspång | Holmsjöhultsängen | 537975 | 6522972 | 1 | 1 | 11 | 1 | SSNC species list. Unknown time. |
| Gnesta | Lilla Ängen | 625628 | 6559735 | 0 | 0 | 1 | 1 | Complementary inventory by N. Hedberg (Stockholm University) 2011. |
| Gnesta | Stora Ängen | 625628 | 6559735 | 0 | 0 | 1 | 1 | Complementary inventory by N. Hedberg (Stockholm University) 2011. Species present in 10 1 m^2^ quadrats plus walk to find additional species. |
| Göteborg | Skändla | 317633 | 6408538 | 1 | 1 | 1 | 1 | Artportalen. Inventory of meadow for regional plant atlas 1994. |
| Gotland | Hörsne Prästänge | 715765 | 6386353 | 1 | 1 | 3 | 1 | SSNC inventory 1989-92 plus additional plants noted since. |
| Habo | Tålåsen | 438439 | 6423641 | 1 | 1 | 3 | 1 | Local plant atlas. Species present in km^2^ containing meadow. |
| Hallsberg | Broby äng | 496434 | 6547333 | 1 | 1 | 4 | 1 | SSNC inventory 2011. |
| Halmstad | Björkelund | 383353 | 6286240 | 1 | 0 | 12 | 1 | SSNC inventory 2002. |
| Hudiksvall | Håsta ängen | 609927 | 6844491 | 0 | 0 | 5 | 1 | SSNC meadow inventory 2004. |
| Karlshamn | Ire | 491857 | 6244787 | 1 | 1 | 2 | 1 | Artportalen. Inventory of meadow by K. Petersson (local botanical group) 2011. |
| Karlskoga | Södra Tolsboda | 488478 | 6580818 | 0 | 0 | 7 | 0 |  |
| Karlstad | I2 ängen | 411987 | 6585165 | 0 | 0 | 3 | 0 |  |
| Katrineholm | Lisstorp | 577656 | 6528234 | 1 | 1 | 5 | 1 | SSNC inventory 1998-2002. |
| Lidingö | Elfvik | 684935 | 6585740 | 0 | 0 | 6 | 1 | SSNC inventory 1998-2001 |
| Lidingö | Södergarn | 682174 | 6586512 | 0 | 0 | 3 | 0 |  |
| Lidköping | Österplana vall | 406794 | 6492701 | 1 | 1 | 9 | 1 | SSNC inventory 2011. |
| Linköping | Bjärka-Säby | 543597 | 6458147 | 1 | 1 | 4 | 1 | SSNC inventory 2012. |
| Motala | Soldatängen | 504025 | 6491788 | 1 | 1 | 4 | 1 | SSNC inventory 2011. |
| Nedre Härjedalen | Stortävremsvallen | 458853 | 6891669 | 0 | 0 | 3 | 0 |  |
| Norrtälje | Färsna | 707217 | 6632154 | 1 | 0 | 4 | 0 |  |
| Nynäshamn | Pettsons hage | 662342 | 6545006 | 1 | 0 | 7 | 1 | SSNC inventory 2011. |
| Öland | Pumpbacken | 585580 | 6267016 | 1 | 0 | 8 | 1 | SSNC inventory 2001-2010. |
| Olofström | Komperskulla | 477922 | 6239354 | 1 | 1 | 6 | 1 | Artdatabanken. Inventory of meadow by B. Nilsson (local botanical group) 2010-11. |
| Olofström | Mulatorp | 468192 | 6252881 | 1 | 1 | 4 | 1 | Artdatabanken. Inventory of meadow by I. Björegren (local botanical group) 2010-11. |
| Skövde | Hökaberg | 421771 | 6481710 | 1 | 1 | 9 | 1 | SSNC inventory 2008-2010 |
| Söderåsen | Ekbacken | 377097 | 6214580 | 1 | 1 | 1 | 0 |  |
| Sollentuna | Vaxmora äng | 666326 | 6595555 | 0 | 0 | 2 | 1 | Local plant atlas. Species in 0.25 km^2^ square containing meadow. 1993. |
| Stockholm | Ekudden | 679620 | 6572787 | 1 | 0 | 3 | 1 | SSNC inventory 1992-2010. |
| Tidaholm | Lammevad | 430159 | 6447525 | 1 | 1 | 6 | 1 | Artportalen. Inventory of meadow for regional plant atlas 1985. |
| Timrå | Torsbodaängen | 627729 | 6934841 | 1 | 1 | 6 | 1 | From regional plant atlas. Species in 0.25 km^2^ square containing meadow. |
| Trollhättan | Grinnsjö | 350199 | 6466012 | 1 | 1 | 9 | 1 | SSNC inventory 2001. |
| Uddevalla | Kurödsängen | 322933 | 6472603 | 1 | 1 | 7 | 1 | From regional plant atlas. Species in 0.05 km^2^ square containing meadow. |
| Ulricehamn | Duvered | 412260 | 6407449 | 1 | 0 | 2 | 0 |  |
| Upplands Väsby | Calmare hage | 664817 | 6604518 | 1 | 0 | 1 | 1 | Complementary inventory by N. Hedberg (Stockholm University) 2011. Species present in 10 1 m^2^ quadrats plus walk to find additional species. |
| Vara | Ranahult | 390267 | 6443417 | 1 | 1 | 10 | 1 | SSNC inventory 2010-2011 |
| Värnamo | Rusarebo | 449887 | 6333666 | 1 | 1 | 5 | 1 | Artportalen. Inventory of meadow for regional plant atlas 1986. |
| Västerås | Rudöklippan | 582295 | 6597589 | 1 | 1 | 9 | 1 | SSNC inventory 2010 |
| Växjö | Tussudden | 493528 | 6307086 | 1 | 1 | 5 | 1 | SSNC inventory, 2000s. |
