# Supplementary material for: Humans as Long-Distance Dispersers of Rural Plant Communities
Source: PLoS One. 2013 May 2;8(5):e62763. doi: 10.1371/journal.pone.0062763 (PMC3642177; doi:10.1371/journal.pone.0062763)
Supplement: Appendix S2 — Species of seeds dispersed by humans from meadows across Sweden. (DOC) [file pone.0062763.s002.doc]

**Appendix S2.** Species of seeds dispersed by humans from meadows across Sweden. Colours represent species considered protected (PRO - Swedish law statute SFS 2007:845), red-listed (VN=Vulnerable, EN=Endangered - [1]) and nationally invasive (NOBANIS. Available: [http://www.nobanis.org](http://www.nobanis.org/). Accessed: 2011 Mar 28). * denotes species considered invasive elsewhere internationally (CABI. Available: <http://www.cabi.org/isc/>. Accessed: 2012 Nov 21). Nomenclature follows Karlsson [2].

| Species (n = 197) | Number of seeds | | Number of meadows where seeds found (n meadows=48) | | Number of meadows where plant registered (n meadows = 36) | | notes |
| --- | --- | --- | --- | --- | --- | --- | --- |
| Achillea millefolium | | 10 | | 5 | 30 |  | |
| Aegopodium podagraria | | 62 | | 9 | 8 |  | |
| Agrimonia eupatoria | | 3 | | 1 | 5 |  | |
| Agrostis sp | | 2258 | | 41 | 27 |  | |
| Alchemilla sp | | 14 | | 5 | 29 |  | |
| Alnus glutinosa* | | 8 | | 3 | 12 |  | |
| Alopecurus pratensis | | 96 | | 9 | 17 |  | |
| Anagallis arvensis* | | 44 | | 6 | 0 |  | |
| Anemone hepatica* (PRO) | | 10 | | 2 | 21 |  | |
| Anemone nemorosa | | 49 | | 1 | 24 |  | |
| Anethum graveolens | | 1 | | 1 | 0 |  | |
| Angelica sylvestris | | 6 | | 1 | 7 |  | |
| Anthoxanthum odoratum | | 108 | | 25 | 30 |  | |
| Anthriscus sylvestris | | 432 | | 28 | 31 |  | |
| Aquilegia vulgaris* | | 35 | | 5 | 6 |  | |
| Arabis hirsuta | | 1 | | 1 | 4 |  | |
| Arenaria serpyllifolia | | 6 | | 4 | 5 |  | |
| Arrhenatherum elatius | | 84 | | 13 | 6 |  | |
| Artemisia sp | | 7 | | 4 | 6 |  | |
| Asperula tinctoria* | | 1 | | 1 | 0 |  | |
| Betula sp | | 2140 | | 43 | 26 |  | |
| Brassica napus | | 1 | | 1 | 0 |  | |
| Briza media* | | 10 | | 5 | 27 |  | |
| Bromopsis inermis | | 2 | | 2 | 1 |  | |
| Bromus sp | | 3 | | 3 | 4 |  | |
| Calamagrostis canescens | | 30 | | 2 | 7 |  | |
| Calamagrostis purpurea | | 380 | | 10 | 1 |  | |
| Calamagrostis stricta | | 47 | | 4 | 0 |  | |
| Callitriche sp* | | 2 | | 1 | 2 | C. stagnalis internationally invasive | |
| Calluna vulgaris | | 7 | | 2 | 12 |  | |
| Campanula sp* | | 279 | | 17 | 34 | C. patula and C. rotundifolia internationally invasive | |
| Carex acuta* | | 17 | | 1 | 3 |  | |
| Carex agg | | 128 | | 29 | 24 | Carex seeds not otherwise represented as a separate “species”. | |
| Carex canescens | | 5 | | 3 | 3 |  | |
| Carex diandra | | 5 | | 2 | 1 |  | |
| Carex distans group | | 16 | | 3 | 6 | C. distans, C. exstensa,C. hostiana, C. punctata, | |
| Carex disticha group | | 104 | | 13 | 15 | C. arenaria, C. disticha, C. echinata, C. ovalis | |
| Carex divulsa | | 10 | | 3 | 1 |  | |
| Carex flava | | 18 | | 2 | 3 |  | |
| Carex nigra group* | | 67 | | 8 | 13 | C. caespitosa, C. elata, C. nigra* | |
| Carex pallescens | | 521 | | 22 | 20 |  | |
| Carex panicea* | | 9 | | 4 | 19 |  | |
| Carex pilulifera | | 32 | | 2 | 12 |  | |
| Carex pulicaris (VU) | | 12 | | 4 | 7 |  | |
| Carex remota group* | | 89 | | 9 | 11 | C. cuprina, C. dioica, C. remota, C. spicata, C. vulpina* | |
| Carex sylvatica | | 91 | | 2 | 2 |  | |
| Carex viridula* | | 29 | | 4 | 3 |  | |
| Centaurea cyanus | | 4 | | 4 | 0 |  | |
| Centaurea jacea* | | 8 | | 3 | 20 |  | |
| Cerastium sp | | 125 | | 26 | 21 |  | |
| Chelidonium majus | | 5 | | 2 | 2 |  | |
| Chenopodium sp | | 8 | | 5 | 2 |  | |
| Cirsium arvense* | | 5 | | 3 | 12 |  | |
| Cirsium oleraceum* | | 1 | | 1 | 0 |  | |
| Cirsium palustre* | | 1 | | 1 | 15 |  | |
| Convolvulus arvensis* | | 1 | | 1 | 1 |  | |
| Crataegus sp | | 2 | | 2 | 13 |  | |
| Crepis sp* | | 65 | | 14 | 12 | C. biennis and C. capillaris internationally invasive | |
| Cynosurus cristatus | | 222 | | 7 | 6 |  | |
| Dactylis glomerata | | 492 | | 28 | 33 |  | |
| Danthonia decumbens* | | 34 | | 9 | 15 |  | |
| Daucus carota | | 1 | | 1 | 0 |  | |
| Deschampsia cespitosa* | | 755 | | 24 | 21 |  | |
| Deschampsia flexuosa | | 353 | | 27 | 21 |  | |
| Dianthus sp | | 64 | | 6 | 11 |  | |
| Digitalis purpurea | | 1 | | 1 | 2 |  | |
| Echium vulgare | | 4 | | 1 | 2 |  | |
| Elymus caninus | | 1 | | 1 | 2 |  | |
| Elytrigia repens | | 1 | | 1 | 10 |  | |
| Epilobium sp | | 70 | | 2 | 18 |  | |
| Fallopia convolvulus | | 2 | | 1 | 2 |  | |
| Festuca sp* | | 1162 | | 44 | 28 | F. heterophylla internationally invasive | |
| Filipendula ulmaria* | | 50 | | 9 | 22 |  | |
| Filipendula vulgaris* | | 177 | | 8 | 15 |  | |
| Fraxinus excelsior (VU) | | 1 | | 1 | 19 |  | |
| Galeopsis bifida/tetrahit | | 10 | | 2 | 10 |  | |
| Galium album* | | 1 | | 1 | 16 |  | |
| Galium aparine | | 6 | | 3 | 2 |  | |
| Galium boreale | | 1 | | 1 | 23 |  | |
| Galium palustre* | | 9 | | 1 | 9 |  | |
| Galium saxatile* | | 7 | | 2 | 4 |  | |
| Galium spurium* | | 1 | | 1 | 0 |  | |
| Galium uliginosum* | | 9 | | 6 | 10 |  | |
| Galium verum | | 179 | | 15 | 25 |  | |
| Gentianella campestris (PRO, EN) | | 9 | | 3 | 4 |  | |
| Geranium robertianum* | | 1 | | 1 | 12 |  | |
| Geranium sylvaticum* | | 2 | | 2 | 18 |  | |
| Geum rivale/urbanum* | | 612 | | 28 | 33 | G. urbanum internationally invasive | |
| Glechoma hederacea | | 1 | | 1 | 8 |  | |
| Helictotrichon pubescens* | | 2 | | 2 | 15 |  | |
| Heracleum mantegazzianum* | | 11 | | 3 | 1 |  | |
| Heracleum sphondylium | | 1 | | 1 | 8 |  | |
| Hieracium sp | | 228 | | 14 | 26 |  | |
| Holcus lanatus* | | 358 | | 23 | 8 |  | |
| Holcus mollis* | | 21 | | 4 | 6 |  | |
| Hypericum agg | | 4 | | 2 | 0 | Hypericum seeds not otherwise represented as a separate “species”. | |
| Hypericum maculatum* | | 123 | | 6 | 26 |  | |
| Hypericum perforatum | | 15 | | 5 | 14 |  | |
| Hypochoeris radicata | | 32 | | 2 | 5 |  | |
| Jasione montana* | | 9 | | 1 | 2 |  | |
| Juncus sp* | | 5949 | | 33 | 20 | J. compressus internationally invasive | |
| Knautia arvensis* | | 51 | | 2 | 19 |  | |
| Lapsana communis* | | 3 | | 2 | 11 |  | |
| Lathyrus linifolius | | 2 | | 1 | 24 |  | |
| Lathyrus pratensis* | | 3 | | 1 | 25 |  | |
| Leontodon sp | | 3 | | 3 | 17 |  | |
| Lepidium sp | | 53 | | 11 | 0 |  | |
| Leucanthemum vulgare | | 292 | | 20 | 31 |  | |
| Linum catharticum | | 106 | | 13 | 11 |  | |
| Linum usitatissimum | | 4 | | 4 | 0 |  | |
| Lolium multiflorum* | | 2 | | 1 | 0 |  | |
| Lolium perenne* | | 17 | | 5 | 4 |  | |
| Lotus corniculatus | | 1 | | 1 | 24 |  | |
| Lychnis flos-cuculi | | 19 | | 3 | 10 |  | |
| Malus sylvestris | | 3 | | 1 | 9 |  | |
| Matricaria recutita | | 32 | | 8 | 1 |  | |
| Medicago lupulina | | 483 | | 9 | 4 |  | |
| Medicago sativa | | 2 | | 1 | 1 |  | |
| Melampyrum arvense | | 1 | | 1 | 1 |  | |
| Melampyrum pratense | | 9 | | 6 | 17 |  | |
| Melica nutans | | 1 | | 1 | 22 |  | |
| Melilotus albus* | | 4 | | 3 | 0 |  | |
| Milium effusum | | 1 | | 1 | 4 |  | |
| Molinia caerulea | | 9 | | 1 | 10 |  | |
| Mycelis muralis* | | 6 | | 2 | 12 |  | |
| Myosotis sp | | 37 | | 12 | 16 |  | |
| Nepeta cataria (PRO, EN) | | 2 | | 1 | 0 |  | |
| Papaver rhoeas/dubium* | | 11 | | 7 | 1 | P. dubium internationally invasive | |
| Pastinaca sativa | | 1 | | 1 | 1 |  | |
| Persicaria lapathifolia | | 2 | | 2 | 3 |  | |
| Persicaria maculosa | | 1 | | 1 | 1 |  | |
| Phalaris arundinacea* | | 90 | | 3 | 0 |  | |
| Phleum arenarium* (EN) | | 3 | | 1 | 0 |  | |
| Phleum pratense | | 88 | | 17 | 24 |  | |
| Pimpinella saxifraga* | | 30 | | 1 | 25 |  | |
| Pinus sylvestris | | 1 | | 1 | 14 |  | |
| Plantago lanceolata* | | 359 | | 19 | 27 |  | |
| Plantago major* | | 75 | | 18 | 13 |  | |
| Plantago media* | | 1 | | 1 | 7 |  | |
| Poa sp* | | 981 | | 41 | 28 | P. annua internationally invasive | |
| Polygala vulgaris | | 1 | | 1 | 18 |  | |
| Polygonum aviculare* | | 7 | | 6 | 3 |  | |
| Potentilla agg | | 121 | | 23 | 17 | Potentilla seeds not otherwise represented as a separate “species”. | |
| Potentilla erecta* | | 84 | | 15 | 25 |  | |
| Primula veris (PRO) | | 67 | | 3 | 26 |  | |
| Prunella vulgaris* | | 55 | | 4 | 21 |  | |
| Prunus spinosa | | 1 | | 1 | 12 |  | |
| Ranunculus acris group | | 325 | | 32 | 31 | R. acris, R. bulbosus, R. flammula, R. repens. | |
| Ranunculus agg | | 1 | | 1 | 23 | Ranunculus seeds not otherwise represented as a separate “species”. | |
| Rhinanthus minor* | | 457 | | 16 | 18 |  | |
| Rhinanthus serotinus | | 21 | | 3 | 3 |  | |
| Rosa sp | | 2 | | 2 | 25 |  | |
| Rubus idaeus | | 33 | | 6 | 24 |  | |
| Rubus subgen Rubus | | 8 | | 3 | 3 |  | |
| Rumex acetosa | | 65 | | 17 | 29 |  | |
| Rumex acetosella | | 25 | | 11 | 16 |  | |
| Rumex agg | | 6 | | 4 | 11 | Rumex seeds not otherwise represented as a separate “species”. | |
| Rumex maritimus | | 1 | | 1 | 0 |  | |
| Sagina nodosa | | 3 | | 2 | 1 |  | |
| Saxifraga granulata* | | 2 | | 1 | 11 |  | |
| Scleranthus annuus | | 1 | | 1 | 5 |  | |
| Scorzonera humilis | | 1 | | 1 | 18 |  | |
| Secale cereale | | 1 | | 1 | 0 |  | |
| Sedum sp | | 1 | | 1 | 9 |  | |
| Senecio sp* | | 46 | | 4 | 10 | S. sarracenicus internationally invasive | |
| Silene dioica* | | 6 | | 1 | 4 |  | |
| Silene nutans | | 7 | | 3 | 6 |  | |
| Silene vulgaris/latifolia* | | 41 | | 2 | 5 | S. vulgaris internationally invasive | |
| Sisymbrium officinale | | 1 | | 1 | 0 |  | |
| Solanum nigrum* | | 1 | | 1 | 2 |  | |
| Sonchus sp | | 5 | | 3 | 5 |  | |
| Spergula arvensis* | | 5 | | 5 | 2 |  | |
| Stachys sp | | 2 | | 1 | 8 |  | |
| Stellaria graminea* | | 98 | | 12 | 26 |  | |
| Stellaria media* | | 16 | | 8 | 6 |  | |
| Succisa pratensis | | 4 | | 1 | 19 |  | |
| Tanacetum vulgare* | | 10 | | 2 | 9 |  | |
| Taraxacum sp | | 22 | | 11 | 27 |  | |
| Thymus serpyllum* | | 7 | | 1 | 5 |  | |
| Tragopogon pratensis | | 12 | | 2 | 11 |  | |
| Trifolium arvense | | 25 | | 5 | 8 |  | |
| Trifolium campestre | | 20 | | 10 | 3 |  | |
| Trifolium dubium | | 2 | | 2 | 1 |  | |
| Trifolium hybridum | | 6 | | 1 | 6 |  | |
| Trifolium incarnatum | | 1 | | 1 | 0 |  | |
| Trifolium pratense | | 146 | | 13 | 29 |  | |
| Trifolium repens | | 56 | | 11 | 27 |  | |
| Tripleurospermum maritimum | | 159 | | 1 | 0 |  | |
| Tripleurospermum perforatum | | 8 | | 1 | 2 |  | |
| Triticum aestivum | | 2 | | 2 | 0 |  | |
| Urtica dioica/urens | | 884 | | 15 | 20 |  | |
| Vaccinium myrtillus | | 2 | | 1 | 20 |  | |
| Valeriana officinalis | | 1 | | 1 | 7 |  | |
| Veronica sp* | | 54 | | 23 | 35 | V. chamaedrys internationally invasive | |
| Vicia cracca | | 1 | | 1 | 28 |  | |
| Viola sp | | 78 | | 13 | 28 |  | |
| Vulpia sp | | 43 | | 3 | 0 |  | |
| TOTAL SEEDLINGS | | 24 354 | | - | - |  | |

**References**

1. Aronsson M, Edqvist M, Andersson U-B, Bertilsson A, Ericsson S, et al. (2010) Karlvaxter – vascular plants (Tracheophyta). The 2010 Red List of Swedish Species (ed U. Gardenfors). Uppsala: ArtDatabanken. pp. 201–221.

2. Karlsson T (1997) Förteckning över svenska kärlväxter [The vascular plants of Sweden - a checklist]. Svensk Botanisk Tidskrift 91: 241–560.
