# Supplementary material for: Humans as Long-Distance Dispersers of Rural Plant Communities
Source: PLoS One. 2013 May 2;8(5):e62763. doi: 10.1371/journal.pone.0062763 (PMC3642177; doi:10.1371/journal.pone.0062763)
Supplement: Appendix S3 — Plant species recorded in meadows but of which no seeds were dispersed. (DOC) [file pone.0062763.s003.doc]

**Appendix S3.** Plant species recorded in meadows but of which no seeds were dispersed. Nomenclature follows Karlsson [1].

**Meadow species where no seeds were found (number of meadows):**

Abies alba (1), Acer platanoides (19), Achillea ptarmica (10), Aconitum lycoctonum (1), Actaea spicata (8), Adoxa moschatellina (1), Aethusa cynapium (1), Agrostemma githago (1), Ajuga pyramidalis (16), Alisma plantago-aquatica (5), Alliaria petiolata (1), Allium oleraceum (9), Allium schoenoprasum (1), Allium scorodoprasum (1), Allium ursinum (2), Allium vineale (1), Alnus incana (2), Alopecurus geniculatus (4), Alyssum alyssoides (2), Amelanchier spicata (2), Anchusa officinalis (1), Androsace septentrionalis (1), Anemone pulsatilla (3), Anemone ranunculoides (2), Angelica archangelica (1), Antennaria dioica (11), Anthemis arvensis (1), Anthemis tinctoria (2), Anthyllis vulneraria (3), Arabidopsis thaliana (12), Arabis glabra (2), Arctium minus (1), Arctium tomentosum (1), Arenaria gothica (1), Argentina anserina (8), Arnica montana (11), Astragalus glycyphyllos (1), Atriplex patula (1), Ballota nigra (1), Barbarea vulgaris (10), Bellis perennis (4), Berberis vulgaris (7), Bidens tripartita (3), Bistorta vivipara (11), Brachypodium pinnatum (1), Brassica rapa (2), Bunias orientalis (1), Butomus umbellatus (1), Calamagrostis arundinacea (12), Calamagrostis epigejos (4), Caltha palustris (9), Calystegia sepium (2), Capsella bursa-pastoris (3), Caragana arborescens (1), Cardamine amara (2), Cardamine bulbifera (2), Cardamine hirsuta (1), Cardamine pratensis (12), Cardaminopsis arenosa (2), Carduus crispus (2), Carex lepidocarpa (3), Carlina vulgaris (1), Carum carvi (6), Centaurea scabiosa (7), Chaenorhinum minus (1), Chrysosplenium alternifolium (3), Cichorium intybus (2), Cicuta virosa (2), Circaea alpina (1), Cirsium acaule (3), Cirsium helenioides (3), Cirsium vulgare (13), Coeloglossum viride (1), Comarum palustre (8), Convallaria majalis (24), Conyza canadensis (1), Cornus alba (1), Corydalis intermedia (2), Corylus avellana (17), Cotoneaster scandinavicus (1), Cuscuta europaea (1), Cynoglossum officinale (1), Daphne mezereum (7), Descurainia sophia (3), Drosera anglica (1), Drosera intermedia (1), Drosera rotundifolia (1), Drymocallis rupestris (2), Echinochloa frumentacea (1), Eleocharis mamillata (1), Eleocharis palustris (2), Eleocharis quinqueflora (1), Empetrum nigrum (2), Eranthis hyemalis (1), Erica tetralix (1), Erigeron acer (2), Eriophorum angustifolium (6), Eriophorum latifolium (2), Eriophorum vaginatum (3), Erodium cicutarium (2), Erophila verna (12), Erysimum cheiranthoides (1), Euonymus europaeus (1), Euphorbia cyparissias (1), Euphorbia helioscopia (1), Euphrasia nemorosa (1), Euphrasia stricta (7), Fagus sylvatica (2), Fallopia dumetorum (1), Fallopia japonica (1), Fragaria moschata (1), Fragaria vesca (28), Fragaria viridis (3), Frangula alnus (15), Fumaria officinalis (4), Gagea lutea (8), Gagea spathacea (1), Galanthus nivalis (1), Galeopsis angustifolia (1), Galeopsis speciosa (3), Galium mollugo (5), Galium suecicum (3), Galium triflorum (1), Genista pilosa (1), Gentiana pneumonanthe (1), Gentianella amarella (1), Geranium bohemicum (1), Geranium columbinum (1), Geranium endressii (1), Geranium molle (2), Geranium pusillum (2), Geranium pyrenaicum (2), Geranium sanguineum (3), Glyceria fluitans (7), Gnaphalium sylvaticum (6), Gnaphalium uliginosum (4), Hedera helix (1), Helianthemum nummularium (5), Helictotrichon pratense (17), Hesperis matronalis (1), Hierochloë odorata (1), Hippophaë rhamnoides (1), Humulus lupulus (2), Hypochoeris maculata (10), Impatiens glandulifera (1), Impatiens parviflora (1), Inula salicina (3), Iris pseudacorus (3), Juniperus communis (23), Lactuca sativa (1), Lactuca serriola (1), Lamium album (1), Lamium purpureum (2), Lamprocapnos spectabilis (1), Larix decidua (1), Laserpitium latifolium (2), Lathraea squamaria (2), Lathyrus niger (2), Lathyrus sylvestris (1), Lathyrus vernus (5), Lemna minor (2), Lilium martagon (2), Linaria vulgaris (6), Lobelia dortmanna (1), Logfia arvensis (1), Lonicera periclymenum (1), Lonicera xylosteum (6), Lupinus polyphyllus (3), Luzula campestris (18), Luzula multiflora (18), Luzula pilosa (18), Lychnis viscaria (14), Lycopus europaeus (4), Lysimachia nummularia (1), Lysimachia thyrsiflora (5), Lysimachia vulgaris (11), Lythrum salicaria (7), Maianthemum bifolium (15), Malus domestica (8), Malva moschata (2), Matricaria matricarioides (5), Melampyrum cristatum (3), Melampyrum nemorosum (5), Melampyrum sylvaticum (12), Mentha arvensis (7), Menyanthes trifoliata (4), Mercurialis perennis (4), Moehringia trinervia (9), Monotropa hypopitys (2), Muscari botryoides (2), Myrica gale (2), Myriophyllum alterniflorum (1), Narcissus poëticus (3), Narcissus pseudonarcissus (2), Nardus stricta (11), Narthecium ossifragum (1), Nuphar lutea (2), Nymphaea alba (2), Odontites vulgaris (1), Ophioglossum vulgatum (1), Origanum vulgare (2), Orthilia secunda (2), Oxalis acetosella (12), Papaver argemone (1), Paris quadrifolia (12), Parnassia palustris (5), Pedicularis palustris (2), Pedicularis sylvatica (1), Persicaria amphibia (4), Persicaria hydropiper (1), Persicaria minor (1), Petasites spurius (1), Peucedanum palustre (6), Phleum phleoides (2), Phragmites australis (5), Picea abies (12), Pilosella aurantiaca (4), Pilosella cymosa (1), Pilosella floribunda (1), Pilosella lactucella (6), Pilosella officinarum (24), Pilosella peleteriana (1), Pinguicula vulgaris (4), Pinus mugo (1), Polemonium caeruleum (1), Polygala amarella (4), Polygonatum multiflorum (2), Polygonatum odoratum (6), Polygonatum verticillatum (1), Populus tremula (21), Potamogeton alpinus (1), Potamogeton natans (3), Primula farinosa (2), Primula polyantha (1), Prunus avium (11), Prunus cerasus (1), Prunus domestica (2), Prunus padus (17), Pulmonaria obscura (1), Pulmonaria officinalis (1), Pyrola chlorantha (1), Pyrola media (1), Pyrola minor (2), Pyrola rotundifolia (5), Pyrus communis (1), Quercus petraea (1), Quercus robur (21), Rhamnus cathartica (6), Rheum rhaponticum (1), Rhynchospora alba (1), Ribes alpinum (5), Ribes nigrum (1), Ribes rubrum (3), Ribes uva-crispa (9), Rorippa amphibia (1), Rorippa palustris (1), Rubus chamaemorus (1), Rubus saxatilis (20), Sagina procumbens (5), Salix aurita (7), Salix caprea (11), Salix cinerea (4), Salix fragilis (2), Salix hastata (1), Salix myrsinifolia (2), Salix pentandra (4), Salix repens (7), Salix viminalis (1), Sambucus nigra (3), Sambucus racemosa (7), Sanicula europaea (2), Saponaria officinalis (1), Satureja acinos (3), Satureja vulgaris (8), Saussurea alpina (1), Saxifraga tridactylites (2), Scabiosa columbaria (1), Schoenoplectus lacustris (2), Schoenoplectus tabernaemontani (1), Schoenus ferrugineus (1), Scirpus sylvaticus (2), Scleranthus perennis (1), Scopolia carniolica (1), Scrophularia nodosa (10), Scutellaria galericulata (6), Selinum carvifolia (2), Serratula tinctoria (1), Sesleria caerulea (2), Silene noctiflora (1), Silene rupestris (2), Sinapis arvensis (2), Solanum dulcamara (3), Solidago canadensis (2), Solidago virgaurea (19), Sorbus aucuparia (27), Sorbus intermedia (15), Sparganium emersum (1), Sparganium erectum (1), Spergula morisonii (1), Spergularia rubra (2), Stellaria alsine (3), Stellaria crassifolia (1), Stellaria holostea (1), Stellaria palustris (2), Symphoricarpos albus (2), Symphytum officinale (1), Syringa vulgaris (3), Tetragonolobus maritimus (1), Thalictrum simplex (2), Thlaspi arvense (2), Thlaspi caerulescens (8), Tilia cordata (10), Torilis japonica (1), Trientalis europaea (8), Trifolium aureum (4), Trifolium badium (1), Trifolium medium (24), Trifolium montanum (2), Triglochin palustre (4), Trollius europaeus (12), Tussilago farfara (11), Typha latifolia (2), Ulmus glabra (5), Ulmus minor (1), Vaccinium oxycoccos (2), Vaccinium uliginosum (6), Vaccinium vitis-idaea (13), Valeriana dioica (1), Valeriana sambucifolia (2), Verbascum nigrum (2), Verbascum thapsus (5), Viburnum opulus (16), Vicia cassubica (1), Vicia hirsuta (1), Vicia pisiformis (1), Vicia sativa (1), Vicia sepium (17), Vicia sylvatica (2), Vicia tetrasperma (5), Vinca minor (4), Vincetoxicum hirundinaria (1).

**Meadow cryptogams (number of meadows):**

Athyrium filix-femina (12) , Blechnum spicant (1) , Botrychium lunaria (9) , Cystopteris fragilis (4) , Dryopteris carthusiana (9) , Dryopteris dilatata (4) , Dryopteris filix-mas (20) , Equisetum arvense (12) , Equisetum fluviatile (7) , Equisetum hyemale (1) , Equisetum palustre (2) , Equisetum pratense (5) , Equisetum sylvaticum (8) , Gymnocarpium dryopteris (8) , Huperzia selago (1) , Lycopodium annotinum (3) , Lycopodium clavatum (2) , Phegopteris connectilis (7) , Polypodium vulgare (12) , Pteridium aquilinum (11) , Selaginella selaginoides (1) , Woodsia ilvensis (1).

**Meadow Orchidaceae (number of meadows):**

Corallorrhiza trifida (1) , Cypripedium calceolus (1) , Dactylorhiza incarnata (4) , Dactylorhiza latifolia (2) , Dactylorhiza maculata (14) , Dactylorhiza majalis (1) , Epipactis helleborine (1) , Epipactis palustris (3) , Gymnadenia conopsea (6) , Listera ovata (9) , Microstylis monophyllos (1) , Neottia nidus-avis (1) , Ophrys insectifera (2) , Orchis mascula (6) , Orchis militaris (1) , Orchis ustulata (1) , Platanthera bifolia (15) , Platanthera chlorantha (16).

**References**

1. Karlsson T (1997) Förteckning över svenska kärlväxter [The vascular plants of Sweden - a checklist]. Svensk Botanisk Tidskrift 91: 241–560.
